# Optimization and validation of sample preparation for metagenomic sequencing of viruses in clinical samples

**DOI:** 10.1186/s40168-017-0317-z

**Published:** 2017-08-08

**Authors:** Dagmara W. Lewandowska, Osvaldo Zagordi, Fabienne-Desirée Geissberger, Verena Kufner, Stefan Schmutz, Jürg Böni, Karin J. Metzner, Alexandra Trkola, Michael Huber

**Affiliations:** 1 Institute of Medical Virology, University of Zurich, Winterthurerstrasse 190, 8057 Zurich, Switzerland; 2 Department of Infectious Diseases and Hospital Epidemiology, University Hospital Zurich, Rämistrasse 100, 8091 Zurich, Switzerland

**Keywords:** Metagenomic sequencing, Clinical samples, Virus diagnostics, Sample preparation

## Abstract

**Background:**

Sequence-specific PCR is the most common approach for virus identification in diagnostic laboratories. However, as specific PCR only detects pre-defined targets, novel virus strains or viruses not included in routine test panels will be missed. Recently, advances in high-throughput sequencing allow for virus-sequence-independent identification of entire virus populations in clinical samples, yet standardized protocols are needed to allow broad application in clinical diagnostics. Here, we describe a comprehensive sample preparation protocol for high-throughput metagenomic virus sequencing using random amplification of total nucleic acids from clinical samples.

**Results:**

In order to optimize metagenomic sequencing for application in virus diagnostics, we tested different enrichment and amplification procedures on plasma samples spiked with RNA and DNA viruses. A protocol including filtration, nuclease digestion, and random amplification of RNA and DNA in separate reactions provided the best results, allowing reliable recovery of viral genomes and a good correlation of the relative number of sequencing reads with the virus input. We further validated our method by sequencing a multiplexed viral pathogen reagent containing a range of human viruses from different virus families. Our method proved successful in detecting the majority of the included viruses with high read numbers and compared well to other protocols in the field validated against the same reference reagent. Our sequencing protocol does work not only with plasma but also with other clinical samples such as urine and throat swabs.

**Conclusions:**

The workflow for virus metagenomic sequencing that we established proved successful in detecting a variety of viruses in different clinical samples. Our protocol supplements existing virus-specific detection strategies providing opportunities to identify atypical and novel viruses commonly not accounted for in routine diagnostic panels.

**Electronic supplementary material:**

The online version of this article (doi:10.1186/s40168-017-0317-z) contains supplementary material, which is available to authorized users.

## Background

To date, sequence-specific PCR is the most common approach for virus identification and quantification in diagnostic laboratories as it is highly sensitive, rapid, and cost effective. However, specific PCR requires prior knowledge of the virus sequence, and a separate assay needs to be designed for each individual virus or virus type. Recently, high-throughput or next generation sequencing (NGS) technologies enabled metagenomic-based identification of viruses by sequencing random fragments of all genomes present in a clinical or environmental sample [1–3]. As viral metagenomics is virus-sequence independent, potentially any virus, cultivable or uncultivable, known or novel, can be readily detected and the method can be applied to all types of virus genomes, including single-stranded DNA and RNA. Several research studies have used this technology in recent years to explore the breadth of the virome in diverse biological and environmental samples including human and animal feces [4–12], blood [13, 14], animal and human tissues [15–17], and human respiratory tract secretions [18–22] and highlighted the validity of the approach to detect rare and novel viruses.

Despite the numerous promising attempts to apply metagenomics to virology, direct sequencing of nucleic acids obtained from biological samples results in a high background of genetic material mainly derived from the host and bacteria hampering the detection of viruses [22, 23]. Sample type greatly influences the composition of sequencing reads, and due to the complexity of clinical materials, sample preparation and virus enrichment methods need to be specifically adapted. Common steps in sample preparation for unbiased metagenomic sequencing are virus enrichment, extraction of nucleic acids, reverse transcription, and unbiased amplification.

Various virus enrichment methods for clinical samples have been suggested. Commonly employed methods include filtration, ultracentrifugation, and nuclease treatment [1, 24–31]. These methods rely on the small size of viruses and the stability of their capsid. Several approaches for virus genome amplification do exist: Multiple displacement amplification (MDA) is often used for whole genome amplification [32–34], e.g., in the VIDISCA method for virus discovery [35]. Linker-amplified shotgun library (LASL) was applied for virus sequencing from marine water samples [8]. The anchored random PCR approach, as used in our study, has been frequently used and described in detail in previous studies [16, 36–42].

Here, we tested various conditions and methods for virus enrichment, nucleic acid extraction, and unbiased amplification with the goal to create a sensitive and robust workflow for metagenomic sequencing of clinical samples in virus diagnostics. We spiked several virus types into plasma from healthy donors to cover different classes of viruses: small non-enveloped DNA (Human adenovirus), large enveloped DNA (Human herpesvirus 4/EBV or Human herpesvirus 5/CMV), small non-enveloped RNA virus (Poliovirus), and enveloped RNA virus (influenzavirus A, Additional file 1: Table S1). We validated our approach using a highly multiplexed viral pathogen reagent containing 25 different viruses. Finally, in experiments with different ratios of spiked viruses, we show a good correlation of the relative number of sequencing reads with the virus input.

## Methods

### Virus stock production

Human adenovirus 7, Human poliovirus 1 (strain LSa), and Human herpesvirus 5 (HHV-5, ATCC VR AD 169) were propagated on MRC-5 cells (human fetal lung fibroblasts) obtained from the European Collection of Cell Cultures (Salisbury, UK). Viruses were cultivated for 14 days, cells were sonicated for 5 s, centrifuged (5 min at 1200 rpm), and the supernatant harvested and filtered (0.45 μm). Human herpesvirus 4 (HHV-4) was propagated on B-95 cells (marmoset B-lymphoblastoid cell line) centrifuged (5 min at 1200 rpm), and the supernatant harvested and filtered (0.45 μm). Influenzavirus A/WSN/33 was propagated on A549 cells (human alveolar basal epithelial cells) and Influenzavirus A/H1N1/PR8 on MDCK cells (canine kidney epithelial cells) for up to 4 days, and the supernatants were centrifuged (10 min at 1200 rpm). Aliquots were stored at −80 °C.

### Virus quantification by quantitative real-time PCR (qPCR)

Viral loads in spiked samples and amplicon concentrations after random amplification were determined by qPCR as described previously for HHV-5 [43], poliovirus [44], adenovirus [45], and influenzavirus (CDC protocol of real-time RT-PCR for swine influenza A H1N1, 28 April 2009). For HHV-4, real-time PCR was performed as described previously [46], but with modified primers (CTTCTCAGTCCAGCGCGTTT and CAGTGGTCCCCCTCCCTAGA) and a modified probe (FAM-CGTAAGCCAGACAGCAGCCAATTGTCAG-TAMRA). All reactions were performed on a ViiA7 Real-Time PCR System (Life Technologies/Thermo Fisher Scientific, Waltham, MA) with the TaqMan RNA-to-Ct 1-Step Kit. One-microliter template (out of 25-μl extraction eluate or 50-μl amplification reaction, respectively) was used with 10-μl master mix, 0.25 μM of each primer, 0.125 μM probe in a total volume of 20 μl. Each sample was tested in duplicates with the following cycling conditions: 30 min at 48 °C, 10 min at 95 °C, 50 cycles of 15 s at 95 °C, and 1 min at 60 °C.

### Absolute virus quantification by digital PCR

For experiments using different ratios of virus input, virus stocks were quantified by digital PCR using the QuantStudio 3D Digital PCR System (Life Technologies/Thermo Fisher Scientific), which allows for absolute quantification without the need of a standard. Reactions were performed in a final volume of 15 μl with 7.5 μl 2× QuantStudio 3D Digital PCR Master Mix, 0.25 μM of each primer and 0.125-μM probe (same primer and probes as used in the qPCR assays described above). The template was diluted as necessary for optimal digital PCR readout. For RNA virus quantification, the reaction additionally contained 0.4 μl 40× TaqMan RT Enzyme Mix and an initial reverse transcription step for 15 min at 48 °C followed by 10 min at 96 °C. Cycling conditions were 50 cycles of 2 min at 60 °C, 30 s at 98 °C, and 60 °C for 2 min. After read-out on the QuantStudio 3D Digital PCR instrument, the raw data was imported into QuantStudio 3D AnalysisSuite Cloud Software (version 3.0.2.2) to calculate absolute copy numbers.

### Virus spike preparation and sample pre-processing

Human clinical samples were obtained from healthy blood donors (Zurich blood donor service, Schlieren, Switzerland) or from diagnostic samples (tested negative for poliovirus, HHV-4, HHV-5, influenzavirus A, and adenovirus) and stored at −20 °C. Samples were centrifuged at 2000 rpm for 10 min (Heraeus Multifuge X3 R, Thermo Fisher Scientific), spiked with viruses to achieve quantitative PCR threshold cycles (ct values) of 22–25, which are considered as high positive samples in diagnostic tests for most viruses. Filtration was done using a 0.45-μm PES filter (TPP, Trasadingen, Switzerland). Freeze-thaw cycles, if applicable, were performed by freezing the samples at −20 °C.

### Nuclease treatment

Nuclease treatment with DNase and RNase was performed as previously described [47] with reagent volumes scaled up for 1000 μl of clinical sample. Briefly, a nuclease mix containing 120 μl DNase (0.92 mg/ml, Roche, Basel, Switzerland), 10 μl RNaseA (0.77 mg/ml, Qiagen, Hilden, Germany), 130 μl 10× nuclease buffer (400 mM Tris-HCl, 100 mM NaCl, 60 mM MgCl_2_, 10 mM CaCl_2_; pH 7.9), 30 μl PBS, and 10-μl water was added. The reaction was incubated for 1 h at 37 °C in a thermoshaker at 1400 rpm. Samples were treated with protease (0.71 mg/ml, Qiagen) for 30 min at 37 °C to remove nuclease activity.

### Nucleic acid extraction

QIAamp Viral RNA Mini Kit (Qiagen), PureLink Viral RNA/DNA Mini Kit (Life Technologies/Thermo Fisher Scientific), and NucliSENS EasyMAG system (BioMérieux, Craponne, France) were used according to the manufacturer’s instructions. Two input volumes of spiked plasma were tested, 500 and 1000 μl, and eluted into 25 μl to achieve a high sample concentration. Large starting volumes were loaded into the extraction columns in multiple steps according to the manufacturer’s instructions, if necessary.

### Unbiased nucleic acid amplification: combined and separate protocols

In a first protocol for unbiased nucleic acid amplification [38], we processed RNA and DNA viruses combined in a single reaction, called here the “combined protocol.” We changed our protocol to include separate amplification steps for RNA and DNA and replace T7 polymerase with DNA Polymerase I Large Klenow Fragment (NEB Biolabs, Ipswich, MA), called the “separate protocol” [9, 42]. For a direct comparison, Klenow fragment was used for both workflows.

For the RNA workflow, cDNA was generated by reverse transcription with a primer containing a random octamer linked to an anchor sequence ATCGTCGTCGTAGGCTGCTCNNNNNNNN [16, 36, 37]. Five microliters of eluate was used as template in a total volume of 20 μl, with 5 μM of random primer, 1 mM of dNTPs, 1× first strand buffer, 20 mM DTT, and 20 U/μl of SuperScript III (Invitrogen/Life Technologies). The template and random primers were heated at 65 °C for 5 min, followed by reverse transcription at 42 °C for 60 min and inactivation at 96 °C for 5 min. Prior to second strand synthesis, cDNA was denatured at 94 °C for 2 min and cooled down to 10 °C for 5 min. The second strand was synthesized with 5 U/μl DNA Polymerase I (Klenow) in 10× NEB buffer in a final volume of 10 μl, at 37 °C for 30 min followed by an enzyme inactivation step at 75 °C for 20 min. An additional step of second strand synthesis used in the initial, combined protocol was omitted.

The DNA workflow started at the denaturation step at 94 °C for 2 min and was performed with the same random primer as used in the RNA workflow prior to second strand synthesis. Second strand synthesis was performed using the same conditions as described for the RNA workflow. Further amplification with the anchor primer and AmpliTaq Gold (Thermo Fisher Scientific) was performed as previously described [38], but separately for the RNA and DNA workflow.

### High-throughput sequencing and bioinformatic analysis

The quality and size of the anchor PCR products were assessed by capillary gel electrophoresis (Fragment Analyzer, Advanced Analytical, Ames, IA). PCR products were quantified with PicoGreen (Invitrogen/Thermo Fisher Scientific) and diluted to 0.2 ng/μl. DNA and RNA preparations were pooled in equal concentrations for constructing sequencing libraries with the NexteraXT protocol (Illumina, San Diego, CA). Individual samples were dual indexed during the library preparation and pooled for sequencing. Libraries were sequenced on a MiSeq (Illumina) for 1 × 150 cycles with version 3 reagents and the “FASTQ only” workflow. Samples were demultiplexed using MiSeq Reporter v2.4.60. Raw sequencing reads are available from the Zenodo repository (10.5281/zenodo.814807). Reads were processed with a dedicated bioinformatic pipeline “VirMet” version 0.3.3 developed in our laboratory (https://github.com/ozagordi/VirMet/releases/tag/v0.3.3) [38]. Briefly, reads were quality-filtered by removing low quality bases (average PHRED score below 20), reads shorter than 75 bp and reads with low entropy (i.e., consisting mainly of repeats). Read passing quality filters were cleaned from non-viral reads by aligning with STAR [48, 49] against, in this order, human, bacterial, bovine, and canine genomes. Reads not matching any of the above genomes were aligned with BLAST [50] against an in-house viral database that contains approximately 46,000 different virus sequences. For each sequencing read that passed the quality filter, the BLAST hit with lowest *e* value was reported, given the identity was higher than 75%. Reads which did not match genomes used in the cleaning step and did not match viral genomes included in the database were reported as of unknown origin.

### Analysis of virus enrichment

Optimization of the protocol was assessed by comparing the virus amplicon concentrations after random amplification by qPCR, by evaluating the fractions of reads of different taxonomic categories after sequencing, and by counting the absolute number of reads and the fraction of the total quality filtered reads for each individual spiked virus. All sequencing experiments were performed in duplicates or triplicates. Statistical analysis was done in R version 3.3.2 using linear models [51]. Coverage plots for viruses used for spiking were generated by mapping virus reads reported by our VirMet pipeline with smalt (http://www.sanger.ac.uk/science/tools/smalt-0, default thresholds) against the following reference genomes: Human adenovirus 7 (GenBank AY495969.1), Human poliovirus 1 Mahoney (V01149.1), and HHV-5 strain AD169 (X17403.1).

## Results

### Extraction with the NucliSENS EasyMAG resulted in the highest virus concentrations

First, three different methods of total nucleic acid extraction were tested: QIAamp Viral RNA Mini Kit, PureLink Viral RNA/DNA Mini Kit, and the automated NucliSENS EasyMAG system. Plasma from healthy donors was spiked with different viruses (adenovirus, poliovirus, HHV-4, influenzavirus A) and extracted, and the concentration in the eluate determined by qPCR. EasyMAG extraction was most efficient for both RNA viruses, while virus concentrations for DNA viruses were similar to PureLink extraction. The Qiagen extraction kit led to the lowest recovery of viral genomes for all tested viruses (Additional file 1: Figure S1 and Table S2). Thus, the EasyMAG system was selected as standard extraction method for all further experiments.

### Filtration substantially enriches for viruses and decreases non-viral reads

In order to assess the effect of sample preparation on the sensitivity of metagenomic sequencing, we spiked plasma from healthy donors with both RNA and DNA viruses (poliovirus, adenovirus, and HHV-4). All viruses were spiked at a qPCR threshold cycle (ct value) in the range of 22–25. Different orders of sample processing corresponding to different pre-analytical situations were then tested using the virus-spiked plasma: the condition “same day” extraction comprised filtration, extraction, and short-term storage before further processing at −20 °C; “pre-processed” comprised filtration, storage at −80 °C, and later extraction; “archived” samples comprised storage at −20 °C, filtration, and extraction. In all three conditions, each sample was processed filtered as well as non-filtered.

**Fig. 1:**
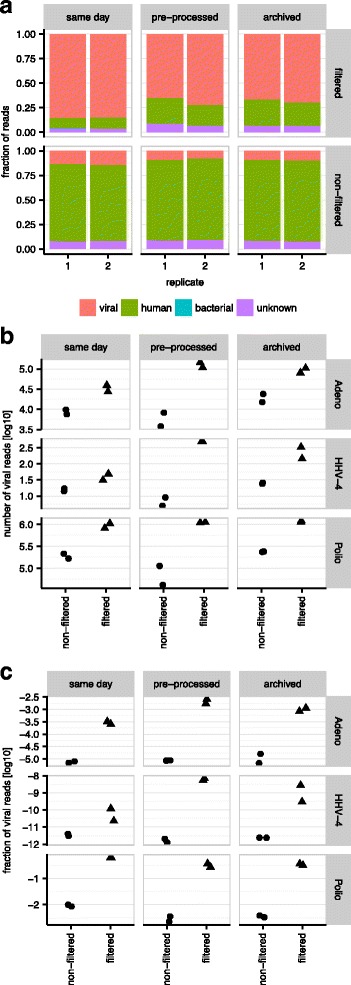
Filtration substantially enriches for virus reads. Plasma samples were spiked with three different viruses (adenovirus, HHV-4, poliovirus) and prepared under the three following conditions: same day comprised filtration, extraction, and freezing at −20 °C; pre-processed comprised filtration, freezing at −80 °C, and extraction; archived included freezing at −20 °C, filtration, and extraction. All conditions were tested with and without filtration. The experiment was performed in duplicates. **a** Distribution of sequencing reads into the taxonomic categories viral, human, bacterial, and unknown origin. **b** Number of reads obtained for each individual virus. **c** Fraction of all quality passing reads obtained for each individual virus

In all conditions, the fraction of virus reads significantly increased as a result of filtration. Same day extraction with filtration and extraction followed by freezing showed the highest enrichment of virus reads (Fig. 1a).

Considering individual viruses used for spiking, for all three viruses, significantly more reads were reported for filtered samples, both in numbers and in the fraction of total reads passing quality filtering (Fig. 1b, c; Additional file 1: Table S3). Conditions pre-processed and archived (including a freezing step before extraction) proved better for DNA viruses (adenovirus and HHV-4) than “same day extraction”. Of note, in all conditions, the highest number of spiked virus reads was reported for poliovirus and the lowest number for HHV-4 (Fig. 1b, c), although the ct values of the input for each virus were similar. For all further samples, we chose pre-processed as standard method.

### Nuclease treatment significantly enriches for virus reads

Next, we tested the effect of nuclease treatment, which takes advantage of the presence of a stable virus capsid that protects the viral genome from digestion, in metagenomic sequencing of plasma spiked with two RNA viruses (poliovirus and influenzavirus A) and two DNA viruses (adenovirus and HHV-4). Spiked plasma incubated at 4 °C without reaction buffer and nucleases served as a control.

**Fig. 2:**
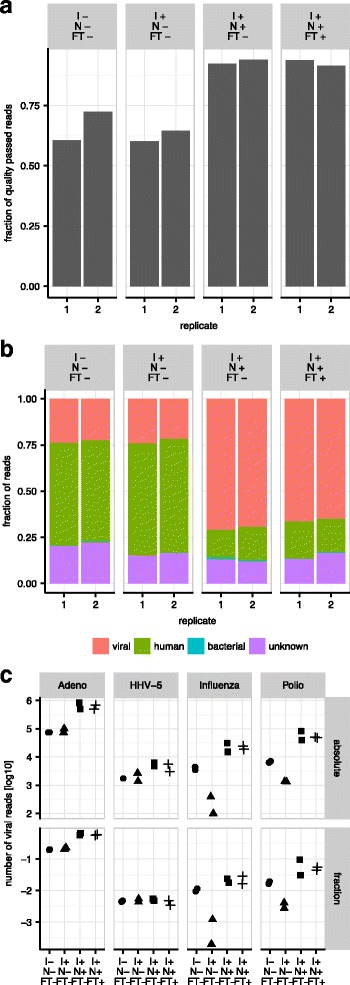
Nuclease treatment significantly enriches for virus reads. To test nuclease treatment in metagenomic sequencing, plasma samples were spiked with four different viruses (adenovirus, HHV-5, influenzavirus, poliovirus) and processed with and without incubation at 37 °C (I +/−), nuclease treatment (N +/−) and freeze-thaw cycles (FT +/−), respectively. The experiment was performed in duplicates. **a** Fraction of sequencing reads that passed quality filtering. **b** Distribution of sequencing reads into the different taxonomic categories viral, human, bacterial, and unknown origin. **c** Number of reads (*upper panels*) and fraction of all quality passing reads (*lower panels*) obtained for each individual virus

In samples that were treated with nucleases, we observed an increase in the fraction of reads passing quality filtering (Fig. 2a, Additional file 1: Table S4). This is a result of the digestion of human DNA containing a lot of repetitive (low entropy) sequences and therefore fewer reads to be removed in quality filtering.

With nuclease treatment, reads of viruses increased significantly, while human background reads decreased (Fig. 2b). Freeze/thaw cycles did not improve virus enrichment by nuclease treatment (Fig. 2b).

Considering sequencing reads from the individual viruses, nuclease treatment enriched all viruses, except the large, enveloped DNA virus HHV-5 (Fig. 2c, Additional file 1: Table S5). Incubation alone, without the enriching effect of nuclease treatment, resulted in a reduced recovery of the RNA viruses influenza and polio.

### Optimization of unbiased nucleic acid amplification

**Fig. 3:**
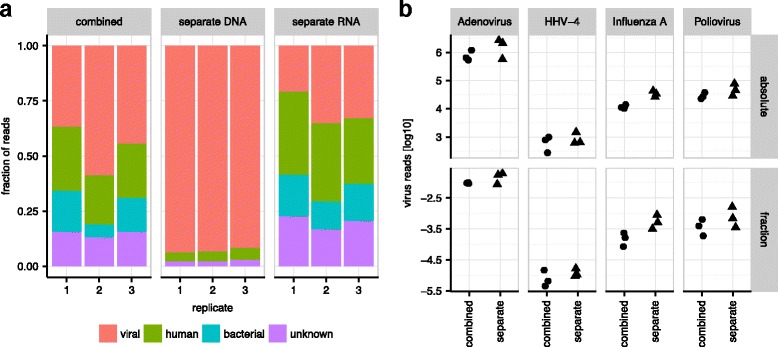
Separate workflows for RNA and DNA yielded higher sequencing reads for DNA viruses. Plasma samples were spiked with four different viruses (adenovirus, HHV-4, influenzavirus, poliovirus) and processed and sequenced with the combined and the new separate workflow. In the separate workflow, random amplification products were pooled before NexteraXT library preparation in equal concentrations. The experiment was performed in triplicates. **a** Distribution of sequencing reads into the different taxonomic categories viral, human, bacterial, and unknown origin. **b** Number of reads (*upper panels*) and fraction of all quality passing reads (*lower panels*) obtained for each individual virus

In order to optimize unbiased amplification of nucleic acids to represent the entire virus population in a sample, we divided the workflow to process RNA and DNA in two separate reactions and changed the enzyme for second strand synthesis from T7 DNA polymerase to Klenow Large Fragment [42]. For comparison, sample volumes were kept identical for both workflows. Using qPCR after random PCR amplification, higher amplicon concentrations were obtained for DNA viruses processed in the separate DNA workflow, when compared to the combined protocol (data not shown). After sequencing, the fraction of virus reads was enriched in the separate protocol for DNA when compared to the combined reaction as well (Fig. 3a). Most importantly, higher numbers of sequencing reads were obtained for all viruses in the separate workflows compared to the combined workflow, especially also for HHV-4 and influenzavirus (Fig. 3b, Additional file 1: Table S6).

As shown before [30], sample preparation can influence the coverage of viral genomes. We therefore aligned virus reads reported by the combined and separate workflow for each virus to its reference genome. Reads were uniformly distributed along the reference genomes in both workflows (Additional file 1: Figure S2).

We further tested if a higher input volume into the reverse transcription reaction and the downstream anchor PCR would increase virus recovery. Using the separate protocol, input strategy 1 used 5 μl of the extract into the reverse transcription reaction and 3 μl of the reverse transcription reaction into the anchor PCR; input strategy 2 used double the amount (10 and 6 μl, respectively). Viral amplicon concentrations after the anchor PCR were higher for input strategy 2 (Additional file 1: Figure S3A). However, the fraction of virus reads and reads assigned to each spiked virus were at similar levels for both input strategies (Additional file 1: Figure S3B and S3C, Additional file 1: Table S7).

**Fig. 4:**
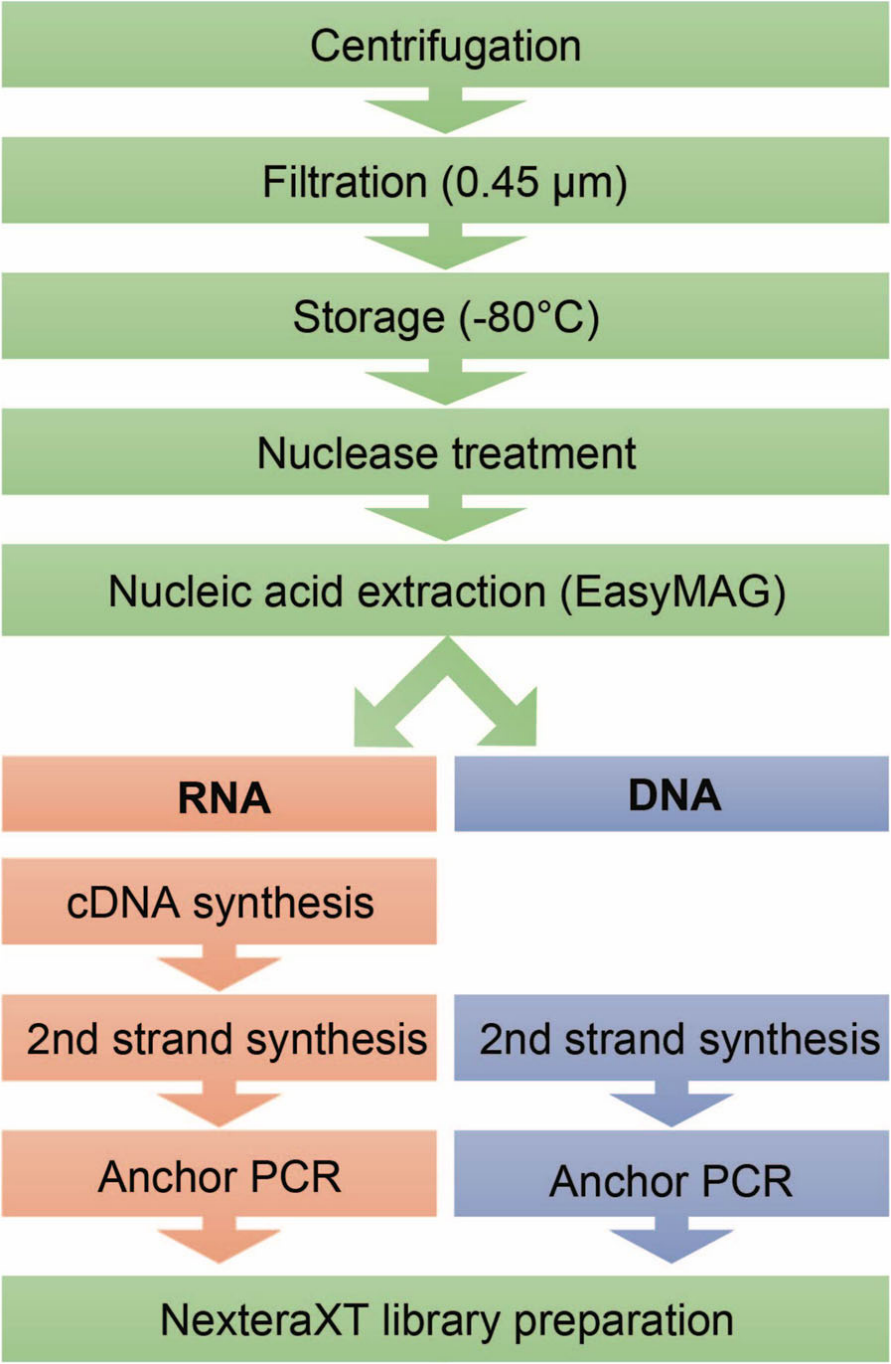
Optimized workflow for metagenomic virus sequencing. A workflow for metagenomic virus sequencing for diagnostic use was developed. Sample pre-processing included low-speed centrifugation, 0.45-μm filtration, storage at −80 °C, and DNase and RNase digestion. Random reverse transcription with an 8N primer including an anchor sequence, second strand synthesis, and anchor PCR amplification was performed separately for an RNA and DNA workflow. The two workflows were pooled in equal concentration for library preparation with NexteraXT

Our final workflow is depicted in Fig. 4.

### The optimized metagenomic sequencing protocol detects the majority of virus species in a highly multiplexed viral pathogen reagent

After establishing a workflow for unbiased, metagenomic sequencing using spiked plasma samples, we used our new protocol for testing a highly multiplexed viral pathogen reagent (11/242-001, National Institute for Biological Standards and Control, South Mimms, UK) containing 25 different human viruses from different virus families [52] (Table 1). We processed and sequenced 1000 μl of the reagent. As our bioinformatic pipeline VirMet determines the taxonomic origin of each individual read, we summed up reads at the species level and noted the most commonly reported strain. Of the 25 viruses expected in the reagent, we detected 17 and 15 different viruses in two replicates, respectively, with high numbers of reads (Table 1). Previous studies have sequenced the same viral pathogen reagent [30, 52]. We randomly subsampled our raw reads to match the numbers analyzed in these studies. Using 2,000,000 reads, we still detected 17 viruses in one of the replicates. Seven of the eight viruses we did not detect (Coronavirus 229E, Norovirus GI and GII, Influenzavirus A and B, Human parainfluenza virus 3) were missed by 15 to 79% of other methods as well [52]. Comparing our results to similar workflows (filtration, nuclease treatment, random amplification, NexteraXT, MiSeq) as, for instance, the sample preparation methods N1-N4 presented in Li et al. [30], our numbers of reads were higher as reported and we identified more virus species (Table 1, 150,000 reads). As previously shown, additional non-targeted viruses are present in the reagent and were detected by us as well (e.g., Bovine viral diarrhea virus, Bocavirus, Enterovirus; Table 1).

**Table 1:** Number of reads reported from sequencing a multiplexed viral pathogen reagent

	Replicate 1			Replicate 2		
Virus species (most frequent strain)	All 3,505,318 reads	2,000,000 reads	150,000 reads	All 2,676,604 reads	2,000,000 reads	150,000 reads
25 target viruses						
Human astrovirus (Human astrovirus 1)	27,622	15,783.2	1184.1	8040.6	596.2	8040.6
Enterovirus B (Human coxsackievirus B4)	27,252	15,539.0	1161.3	22,707.3	1704.4	22,707.3
Human herpesvirus 1	67	39.0	3.5	81.4	5.5	81.4
Human herpesvirus 2	2	1.3	0.4	7.0	0.4	7.0
Human herpesvirus 3 (Human herpesvirus 3 strain Dumas)	34	17.4	1.4	84.2	6.4	84.2
Human herpesvirus 4	166	98.1	8.2	104.7	8.7	104.7
Human herpesvirus 5	8327	4743.1	352.1	7028.5	524.4	7028.5
Human mastadenovirus C (Human adenovirus 2)	23	11.5	0.8	299.1	19.6	299.1
Human mastadenovirus F (Human adenovirus 41)	2	2.0	0.0	13.9	1.7	13.9
Human metapneumovirus	0	0.0	0.0	0.0	0.0	0.0
Human parainfluenza virus 1	21,387	12,170.8	921.2	9601.2	718.6	9601.2
Human parainfluenza virus 2	2879	1651.5	119.6	33.1	1.6	33.1
Human parainfluenza virus 3	0	0.0	0.0	0.0	0.0	0.0
Human parainfluenza virus 4 (Human parainfluenzavirus 4b)	21,858	12,478.6	924.3	10,089.8	760.2	10,089.8
Human respiratory syncytial virus	0	0.0	0.0	229.4	15.5	229.4
Human Rhinovirus A (Human rhinovirus A39)	0	0.0	0.0	2238.4	158.6	2238.4
Influenzavirus A H1N1	0	0.0	0.0	0.0	0.0	0.0
Parechovirus A (Human parechovirus 3)	1,492,756	851,560.6	63,862.7	565,228.5	42,434.5	565,228.5
Rotavirus A	6	2.8	0.3	8.5	0.8	8.5
Sapporovirus (Sapovirus Hu/GI.2/BR-DF01/BRA/2009 and Hu/G1/BE-HPI01/DE/2012)	1019	575.5	45.2	62.8	5.1	62.8
Human coronavirus 229E	0	0.0	0.0	0.0	0.0	0.0
Norovirus GI	0	0.0	0.0	0.0	0.0	0.0
Norovirus GII	0	0.0	0.0	0.0	0.0	0.0
Influenza virus B	0	0.0	0.0	0.0	0.0	0.0
Influenza virus A H3N2	0	0.0	0.0	0.0	0.0	0.0
Non-target viruses						
Bovine viral diarrhea virus 1	1446	820.6	63.6	1891.8	140.3	1891.8
Bovine viral diarrhea virus 2	1	0.4	0.4	0.0	0.0	0.0
Primate bocaparvovirus 1	0	0.0	0.0	0.0	0.0	0.0
Primate bocaparvovirus 2	208	118.2	7.5	91.6	6.8	91.6
Human enterovirus (Enterovirus CA55–1988)	3199	1834.7	140.6	380.6	28.7	380.6
Aichi virus	0	0.0	0.0	0.0	0.0	0.0
Ungulate bocaparvovirus 1	0	0.0	0.0	0.0	0.0	0.0
Porcine/other circovirus	0	0.0	0.0	0.0	0.0	0.0
Total virus reads	1,608,254	917,448.3	68,797.2	840,888	628,222.4	47,138.0
Number of detected target viruses	15	14.4	11.7	17	17.0	15.7

For subsamples of reads, the average number of virus reads and detected target viruses in 10 random samplings is shown

### Ratio of virus reads correlates with concentration of viruses in spiked plasma

In order to confirm that our protocol detects viruses in a reproducible and quantitative manner, we sequenced plasma samples spiked with different ratios and concentrations of an RNA virus (poliovirus or influenzavirus) and a DNA virus (adenovirus). First, we spiked a concentration of 50,000 copies/μl of both viruses; we then spiked ten times more and ten times less of one virus while keeping the other virus constant at 50,000 copies/μl, and vice versa for the other virus.

**Fig. 5:**
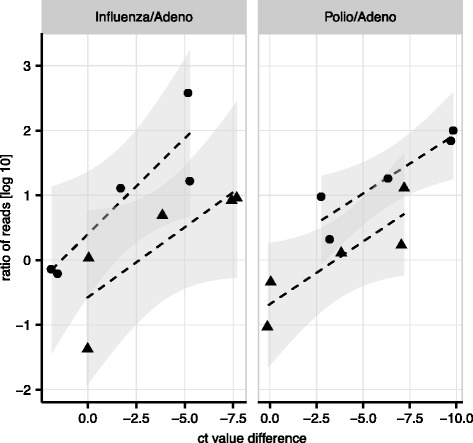
Ratio of virus reads correlates with concentration of viruses in spiked plasma. Poliovirus/adenovirus and influenzavirus/adenovirus were spiked in healthy donor plasma in different concentrations: at the same concentration for both viruses, ten times more of one virus (keeping the other virus constant) and ten times less of one virus (keeping the other virus constant). The ratio of the sequencing reads for each virus combination was correlated with the concentration ratio (ct value difference) in the input sample. Two independent experiments are shown (circles and triangles, respectively). Shaded areas show the 95% confidence interval. *R* ^2^ = 0.74, 0.59, 0.79, and 0.69 and *p*values = 0.04, 0.08, 0.02, and 0.05 for influenza/adenovirus experiments 1 and 2 and polio/adenovirus experiments 1 and 2, respectively

After extraction, viral amplicons were quantified in the eluate and the ratio of spiked viruses was perfectly maintained (Additional file 1: Figure S4). After random amplification and sequencing with the separate workflow, we calculated the ratio of the number of reads of the two viruses, as the number of total reads in each sample varied and the viruses are amplified differentially. The ratio of the virus reads in a sample correlated well with the difference in ct values in the initial sample, showing that our method amplifies and detects both viruses in a reproducible and quantitative manner (Fig. 5).

### Sample preparation of clinical samples other than plasma

**Fig. 6:**
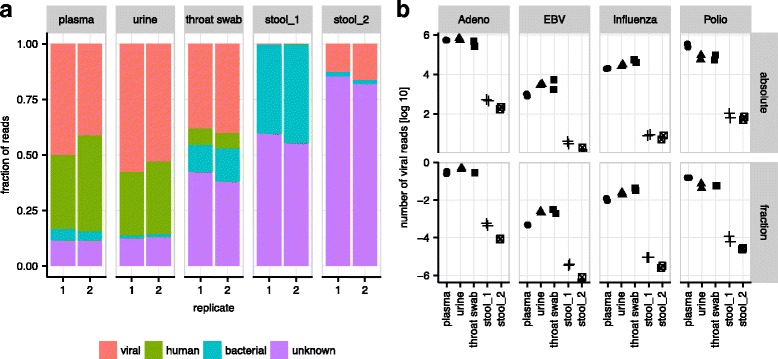
Sequencing of the same virus spike in different clinical samples*.* The same volume of plasma, urine, throat swab, and two different stool samples were spiked with the same amount of four different viruses (adenovirus, HHV-4, influenzavirus, poliovirus) and sequenced. The experiment was done in duplicates. **a** Distribution of sequencing reads into the different taxonomic categories viral, human, bacterial, and unknown origin. **b** Number of reads (*upper panels*) and fraction of all quality passing reads (*lower panels*) obtained for each individual virus

In order to test if our method established with plasma also works for other clinical samples, we spiked the same volume of plasma, urine, throat swab, and two different stool suspensions with the same amount of viruses. After sequencing, the fraction of virus reads in urine and throat swab was similar as for plasma (Fig. 6, Additional file 1: Table S8). For stool samples, however, the amount of unknown background reads was substantially higher and virus reads, if detected, were mostly bacteriophages. For the individual viruses that were spiked, the number and fraction of virus reads were significantly decreased in both stool samples. A more stringent virus enrichment protocol is therefore needed to sequence stool samples to achieve the same sensitivity [53, 54].

## Discussion

In this study, we developed and validated a sample preparation protocol for unbiased amplification and high-throughput metagenomic sequencing of viruses in routine diagnostic use. In an unbiased metagenomic approach, prior knowledge of the virus sequence is not required. In principle, it can therefore detect any virus. However, sample processing needs to be optimized for recovery and amplification of viral genomes that might be present only in very small amounts in clinical samples. We optimized such a protocol using plasma samples spiked with different classes of viruses.

First, we tested filtration, extraction, and nuclease digestion procedures in order to find optimal conditions for virus enrichment and reduction of unwanted, non-viral reads. Filtration proved to be indispensable, as the number of virus reads after sequencing significantly increased. Additionally, sample pre-processing including a freeze thaw cycle showed the best enrichment (Fig. 1), and for best integration into the daily laboratory workflow, we chose pre-processed for further experiments. Two of the extraction methods tested here have been compared before with a higher scoring for the QIAamp Viral RNA Mini Kit compared to the PureLink Viral RNA/DNA Mini Kit [27]. Other studies reported limited coverage of the genome using a magnetic bead-based method similar in principle to the EasyMAG [30]. However, we did not observe this as we obtained good coverage of the entire genome (Additional file 1: Figure S2).

Nuclease treatment, which takes advantage of the stable virus capsid that shields the viral genome from digestion, significantly reduced the fraction of human reads and increased the amount of reads passing the quality filter in our analysis pipeline. This is a result of the digestion of human DNA containing high numbers of repetitive (low entropy) sequences and important for maximizing the amount of quality reads to increase sensitivity. Nuclease treatment enriched for all viruses, except HHV-5, which might, probably due to its size or envelope, not be as stable as smaller viruses. While the combination of low-speed centrifugation, filtration, and nuclease-treatment also showed the greatest increase in the proportion of viral sequences in other studies [29] and filtration and DNase treatment lead to dramatic improvements [25], some studies only found minor differences among the methods with or without filtration and nuclease digestion [30]. The reason for these discrepancies might be that some studies compare pre-sequencing nucleic acid concentrations while others compare sequencing reads. Enrichment methods in general decrease the absolute concentration; however, in our hands, the overall gain of nuclease treatment to increase the ratio of virus over host prevails.

Poliovirus reads were recovered in highest numbers in our experiments, although the amount of spiked viruses was adjusted to similar levels (ct values). This might be due to the fact that poliovirus has the smallest virion and genome size among the spiked viruses, what might have facilitated its efficient enrichment and amplification. In contrast, HHV-4 virus is a large virus in respect to virion and genome size and often only single reads could be detected. It was suggested that filtration might actually have an adverse effect on large virus genomes such as herpesvirus and mimivirus [28]. In other studies of virus profiling in clinical specimens, lower numbers of HHV-4 reads as expected were also reported [55]. However, experiments using HHV-5, which belongs to the same virus family as HHV-4, resulted in many more virus reads. Therefore, it is conceivable that other factors than virus and genome size are influencing the number of recovered viral genomes.

Separating the random amplification into two separate workflows for RNA and DNA was more advantageous for the DNA viruses than for the RNA viruses. This could be either a result of concomitant amplification of DNA viruses in the RNA workflow or an indication that DNA genome amplification was negatively affected during the reverse transcription and second strand synthesis in the previous combined workflow. Yet, for both the combined and separate workflow, reads were uniformly distributed across the reference genomes and we did not observe differences.

In order to validate our method, we sequenced a multiplexed viral pathogen reagent that was expected to contain 25 viruses across different families, genome types, and sizes [52]. At most, we identified 17 different viruses (Table 1). We did not detect any reads for eight viruses supposed to be in the reagent, all of them single-stranded RNA viruses (Coronavirus 229E, Norovirus GI and GII, Influenzavirus A and B, Metapneumovirus, Human Parainfluenzavirus 3). However, most of these viruses were present at very low concentration in the viral pathogen reagent (undetectable by qPCR after mixing of the reagent) and were also frequently not detected by other groups that probed this reagent. In the study by Mee et al. [52], a wide range of different sample volumes and enrichment, amplification, and sequencing methods were used. Stochastic effects in detection of very low abundant viruses and differences in analysis thresholds could have played a role as well. Comparing our results to more similar workflows (filtration, nuclease treatment, random amplification, NexteraXT, MiSeq), our numbers of reads were higher than reported and we identified more virus species [30]. Therefore, we think that our method, which might still have potential for improved sensitivity, shows no bias against a certain virus family or genome type (e.g., ssRNA).

To assess how robust our sample preparation is, we correlated different ratios of spiked viruses with the number of corresponding sequencing reads. In both virus combinations and experiments performed, there was a good correlation between the ratio of spiked viruses and the ratio of the resulting reads (Fig. 4). Direct correlations of viral copies with percentages of sequencing reads have been suggested [18, 54]. However, a direct quantification of input copy numbers based on sequencing reads seems not applicable. Viruses have different properties such as the presence of an envelope, types of genomes, and virion sizes, and certain virus or genome types are amplified preferentially as we have seen with Poliovirus in our experiments. Adding to the complexity, different composition of the genetic background in clinical samples will strongly influence the yield of virus reads. Nevertheless, the correlations showed that the ratio of sequencing reads is preserved over different input ratios and concentrations, signifying that the amplification method itself preserves the relative contribution of different viruses in a sample.

Finally, spiking the same amount of viruses in different samples showed that the protocol presented here could be applied not only for plasma, but also for other clinical samples such as urine and swabs. A more stringent virus enrichment protocol is needed to sequence stool samples or biopsies, as those contain a lot of bacterial or human background reads, respectively [53, 54].

## Conclusions

A metagenomic virus sequencing protocol, as presented here, allows diagnostic laboratories to potentially identify any virus present in clinical samples with a single analysis. Such an approach reduces the time and cost spent today on multiple tests performed for each distinct virus and allows detecting rare or novel viruses not accounted for in routine test panels. Characterization of the virome and its alterations in specific disease settings might help to better understand and manage infectious diseases. Finally, our analysis highlights the need for validation methods and standards for metagenomic sequencing approaches in clinical diagnostics.

## Additional file

Additional file 1:
**Table S1.** Characteristics of viruses used in the virus-spike experiments. **Table S2.** Analysis of variance **Figure S1** (extraction experiments). **Table S3.** Analysis of Variance Fig. 1 (filtration experiments). **Table S4.** Analysis of variance Fig. 2 (nuclease digestion experiments). **Table S5.** Analysis of variance Fig. 2 (nuclease digestion experiments). **Table S6.** Analysis of variance Fig. 3 (separate workflow experiments). **Table S7.** Analysis of variance Figure S3 (input experiments). **Table S8.** Analysis of variance Fig. 6 (other sample types). **Table S9.** Raw sequencing data files (available at Zenodo 10.5281/zenodo.814807). **Figure S1.** Extraction with the NucliSENS EasyMAG resulted in the highest virus concentrations. **Figure S2.** Reads assigned to spiked virus were uniformly distributed along the reference genomes. **Figure S3.** Increasing the PCR input volume had no effect on viral enrichment. **Figure S4.** After extraction, viral amplicons were quantified in the eluate and the ratio of spiked viruses was perfectly maintained. (PDF 540 kb)
