# Correction to: Optimization and validation of sample preparation for metagenomic sequencing of viruses in clinical samples

**DOI:** 10.1186/s40168-017-0351-x

**Published:** 2017-10-12

**Authors:** Dagmara W. Lewandowska, Osvaldo Zagordi, Fabienne-Desirée Geissberger, Verena Kufner, Stefan Schmutz, Jürg Böni, Karin J. Metzner, Alexandra Trkola, Michael Huber

**Affiliations:** 1 Institute of Medical Virology, University of Zurich, Winterthurerstrasse 190, 8057 Zurich, Switzerland; 2 Department of Infectious Diseases and Hospital Epidemiology, University Hospital Zurich, Rämistrasse 100, 8091 Zurich, Switzerland

## Correction

Following publication of the original article [1], the authors were alerted by a colleague of a column duplication in Table 1. Since the summary row was correct, though, the interpretation and the conclusion of the article were not affected

**Table 1:** Number of reads reported from sequencing a multiplexed viral pathogen reagent

	Replicate 1			Replicate 2		
Virus species (most frequent strain)	all 3'505'318 reads	2'000'000 reads	150'000 reads	all 2'676'604 reads	2'000'000 reads	150'000 reads
25 target viruses						
Human astrovirus (Human astrovirus 1)	27'622	15’783.2	1’184.1	10'781	8’040.6	596.2
Enterovirus B (Human coxsackievirus B4)	27'252	15’539.0	1’161.3	30'409	22’707.3	1’704.4
Human herpesvirus 1	67	39.0	3.5	106	81.4	5.5
Human herpesvirus 2	2	1.3	0.4	9	7.0	0.4
Human herpesvirus 3 (Human herpesvirus 3 strain Dumas)	34	17.4	1.4	105	84.2	6.4
Human herpesvirus 4	166	98.1	8.2	149	104.7	8.7
Human herpesvirus 5	8'327	4’743.1	352.1	9'398	7’028.5	524.4
Human mastadenovirus C (Human adenovirus 2)	23	11.5	0.8	404	299.1	19.6
Human mastadenovirus F (Human adenovirus 41)	2	2.0	0.0	19	13.9	1.7
Human metapneumovirus	0	0.0	0.0	0	0.0	0.0
Human parainfluenza virus 1	21'387	12’170.8	921.2	12'897	9’601.2	718.6
Human parainfluenza virus 2	2'879	1651.5	119.6	44	33.1	1.6
Human parainfluenza virus 3	0	0.0	0.0	0	0.0	0.0
Human parainfluenza virus 4 (Human parainfluenzavirus 4b)	21'858	12’478.6	924.3	13'456	10’089.8	760.2
Human respiratory syncytial virus	0	0.0	0.0	298	229.4	15.5
Human Rhinovirus A (Human rhinovirus A39)	0	0.0	0.0	2'983	2’238.4	158.6
Influenzavirus A H1N1	0	0.0	0.0	0	0.0	0.0
Parechovirus A (Human parechovirus 3)	1'492'756	851’560.6	63862.7	756'598	565’228.5	42’434.5
Rotavirus A	6	2.8	0.3	12	8.5	0.8
Sapporovirus (Sapovirus Hu/GI.2/BR-DF01/BRA/2009 and Hu/G1/BE-HPI01/DE/2012)	1'019	575.5	45.2	82	62.8	5.1
Human coronavirus 229E	0	0.0	0.0	0	0.0	0.0
Norovirus GI	0	0.0	0.0	0	0.0	0.0
Norovirus GII	0	0.0	0.0	0	0.0	0.0
Influenza virus B	0	0.0	0.0	0	0.0	0.0
Influenza virus A H3N2	0	0.0	0.0	0	0.0	0.0
Non-target viruses						
Bovine viral diarrhea virus 1	1'446	820.6	63.6	2'526	1’891.8	140.3
Bovine viral diarrhea virus 2	1	0.4	0.4	0	0.0	0.0
Primate bocaparvovirus 1	0	0.0	0.0	0	0.0	0.0
Primate bocaparvovirus 2	208	118.2	7.5	117	91.6	6.8
Human enterovirus (Enterovirus CA55-1988)	3'199	1’834.7	140.6	495	380.6	28.7
Aichi virus	0	0.0	0.0	0	0.0	0.0
Ungulate bocaparvovirus 1	0	0.0	0.0	0	0.0	0.0
Porcine/other circovirus	0	0.0	0.0	0	0.0	0.0
Total virus reads	1'608'254	917'448.3	68'797.2	840'888	628'222.4	47'138.0
Number of detected target viruses	15	14.4	11.7	17	17.0	15.7

For subsamples of reads the average number of virus reads and detected target viruses in 10 random samplings is shown

.
