# Supplementary material for: Optimization and validation of sample preparation for metagenomic sequencing of viruses in clinical samples
Source: Microbiome. 2017 Aug 8;5:94. doi: 10.1186/s40168-017-0317-z (PMC5549297; doi:10.1186/s40168-017-0317-z)
Supplement: Additional file 1: — Table S1. Characteristics of viruses used in the virus-spike experiments. Table S2. Analysis of variance Figure S1 (extraction experiments). Table S3. Analysis of Variance Fig. 1 (filtration experiments). Table S4. Analysis of variance Fig. 2 (nuclease digestion experiments). Table S5. Analysis of variance Fig. 2 (nuclease digestion experiments). Table S6. Analysis of variance Fig. 3 (separate workflow experiments). Table S7. Analysis of variance Figure S3 (input experiments). Table S8. Analysis of variance Fig. 6 (other sample types). Table S9. Raw sequencing data files (available at Zenodo 10.5281/zenodo.814807). Figure S1. Extraction with the NucliSENS EasyMAG resulted in the highest virus concentrations. Figure S2. Reads assigned to spiked virus were uniformly distributed along the reference genomes. Figure S3. Increasing the PCR input volume had no effect on viral enrichment. Figure S4. After extraction, viral amplicons were quantified in the eluate and the ratio of spiked viruses was perfectly maintained. (PDF 540 kb) [file 40168_2017_317_MOESM1_ESM.pdf]

Supplemental Tables

Table S1: Characteristics of viruses used in the virus-spike experiments.

Virus	Envelope	Virion size [nm]	Genome	Genome size [kb]
HHV-4	enveloped	150-200	linear ds DNA	184
HHV-5	enveloped	150-200	linear ds DNA	230
Human Adenovirus	non-enveloped	90	linear ds DNA	36
Influenzavirus A	enveloped	80-120	linear ss RNA(-)	13.5
Human Poliovirus 1	non-enveloped	30	linear ss RNA(+)	7.4

Reference: http://viralzone.expasy.org

Table S2: Analysis of Variance Figure S1 (extraction experiments)

Response: ct value	Df	Sum Sq	Mean Sq	F value	Pr(>F)
Protocol	2	178.2948875	89.14744375	175.4259424	2.74E-19
Virus	3	215.0848229	71.69494097	141.0825937	5.87E-20
Protocol:virus	6	11.45554583	1.909257639	3.757071504	0.005262946
Residuals	36	18.294375	0.508177083	NA	NA

Table S3: Analysis of Variance Figure 1 (filtration experiments)

Response: log10 fraction of reads	Df	Sum Sq	Mean Sq	F value	Pr(>F)
Filtration	1	7.70	7.70	819.49	1.8E-16
Processing	2	0.11	0.05	5.81	1.1E-02
Virus	2	97.27	48.63	5176.09	1.4E-25
Filtration:processing	2	0.37	0.19	19.73	2.9E-05
Filtration:virus	2	0.08	0.04	4.46	2.7E-02
Processing:virus	4	0.34	0.08	8.99	3.6E-04
Filtration: processing:virus	4	0.24	0.06	6.49	2.0E-03
Residuals	18	0.17	0.01	NA	NA

Table S4: Analysis of Variance Figure 2 (nuclease digestion experiments)

Response: fraction of quality passing reads	Df	Sum Sq	Mean Sq	F value	Pr(>F)
Nuclease	3	0.16	0.05	25.80	4.5E-03
Residuals	4	0.01	0.00	NA	NA

Table S5: Analysis of Variance Figure 2 (nuclease digestion experiments)

Response: log10 fraction of reads	Df	Sum Sq	Mean Sq	F value	Pr(>F)
Nuclease	3	3.47	1.16	36.55	2.2E-07
Virus	3	17.23	5.74	181.47	1.5E-12
Nuclease:virus	9	2.44	0.27	8.57	1.2E-04
Residuals	16	0.51	0.03	NA	NA

Table S6: Analysis of Variance Figure 3 (separate workflow experiments)

**Response: log10 fraction of reads**	**Df**	**Sum Sq**	**Mean Sq**	**F value**	**Pr(>F)**
Protocol	1	0.57	0.57	11.45	3.8E-03
Virus	3	28.84	9.61	192.46	9.2E-13
Protocol:virus	3	0.13	0.041	0.84	4.9E-01
Residuals	4	0.80	0.05	NA	NA

Table S7: Analysis of Variance Figure S3 (input experiments)

**Response: log10 fraction of reads**	**Df**	**Sum Sq**	**Mean Sq**	**F value**	**Pr(>F)**
Input	1	0.01	0.01	0.79	4.0E-01
Virus	3	20.40	6.80	490.10	2.1E-09
Input:virus	3	0.03	0.01	0.64	6.1E-01
Residuals	8	0.11	0.01	NA	NA

Table S8: Analysis of Variance Figure 6 (other sample types)

**Response: log10 fraction of reads**	**Df**	**Sum Sq**	**Mean Sq**	**F value**	**Pr(>F)**
Sample	4	92.51	23.13	2890.22	3.7E-26
Virus	3	27.40	9.13	1141.57	1.3E-21
Sample:virus	12	1.67	0.14	17.41	9.4E-08
Residuals	20	0.15	0.01	NA	NA

Table S9: Raw Sequencing Data Files (available at Zenodo 10.5281/zenodo.814807)

Experiment	Condition	Replicate	FASTQ-File
Figure 1	same day filtered	1	Figure_1_same_day_filtered_1.fastq.gz
Figure 1	same day filtered	2	Figure_1_same_day_filtered_2.fastq.gz
Figure 1	same day non-filtered	1	Figure_1_same_day_non-filtered_1.fastq.gz
Figure 1	same day non-filtered	2	Figure_1_same_day_non-filtered_2.fastq.gz
Figure 1	pre-processed filtered	1	Figure_1_pre-processed_filtered_1.fastq.gz
Figure 1	pre-processed filtered	2	Figure_1_pre-processed_filtered_2.fastq.gz
Figure 1	pre-processed non-filtered	1	Figure_1_pre-processed_non-filtered_1.fastq.gz
Figure 1	pre-processed non-filtered	2	Figure_1_pre-processed_non-filtered_2.fastq.gz
Figure 1	archived filtered	1	Figure_1_archived_filtered_1.fastq.gz
Figure 1	archived filtered	2	Figure_1_archived_filtered_2.fastq.gz
Figure 1	archived non-filtered	1	Figure_1_archived_non-filtered_1.fastq.gz
Figure 1	archived non-filtered	2	Figure_1_archived_non-filtered_2.fastq.gz
Figure 2	I− N− FT−	1	Figure_2_I−_N−_FT−_1.fastq.gz
Figure 2	I− N− FT−	2	Figure_2_I−_N−_FT−_2.fastq.gz
Figure 2	I+ N− FT−	1	Figure_2_I+_N−_FT−_1.fastq.gz
Figure 2	I+ N− FT−	2	Figure_2_I+_N−_FT−_2.fastq.gz
Figure 2	I+ N+ FT−	1	Figure_2_I+_N+_FT−_1.fastq.gz
Figure 2	I+ N+ FT−	2	Figure_2_I+_N+_FT−_2.fastq.gz
Figure 2	I+ N+ FT+	1	Figure_2_I+_N+_FT+_1.fastq.gz
Figure 2	I+ N+ FT+	2	Figure_2_I+_N+_FT+_2.fastq.gz
Figure 3	combined	1	Figure_3_combined_1.fastq.gz
Figure 3	combined	2	Figure_3_combined_2.fastq.gz
Figure 3	combined	3	Figure_3_combined_2.fastq.gz
Figure 3	separate DNA	1	Figure_3_separate_DNA_1.fastq.gz
Figure 3	separate DNA	2	Figure_3_separate_DNA_2.fastq.gz
Figure 3	separate DNA	3	Figure_3_separate_DNA_2.fastq.gz
Figure 3	separate RNA	1	Figure_3_separate_RNA_1.fastq.gz
Figure 3	separate RNA	2	Figure_3_separate_RNA_2.fastq.gz
Figure 3	separate RNA	3	Figure_3_separate_RNA_2.fastq.gz
Figure 5	Influenza/Adeno 1:1	1	Figure_5_Influenza_Adeno_1_1_1.fastq.gz
Figure 5	Influenza/Adeno 10:1	1	Figure_5_Influenza_Adeno_10_1_1.fastq.gz
Figure 5	Influenza/Adeno 0.1:1	1	Figure_5_Influenza_Adeno_0.1_1_1.fastq.gz
Figure 5	Influenza/Adeno 1:10	1	Figure_5_Influenza_Adeno_1_10_1.fastq.gz
Figure 5	Influenza/Adeno 1:0.1	1	Figure_5_Influenza_Adeno_1_0.1_1.fastq.gz
Figure 5	Influenza/Adeno 1:1	2	Figure_5_Influenza_Adeno_1_1_2.fastq.gz
Figure 5	Influenza/Adeno 10:1	2	Figure_5_Influenza_Adeno_10_1_2.fastq.gz
Figure 5	Influenza/Adeno 0.1:1	2	Figure_5_Influenza_Adeno_0.1_1_2.fastq.gz
Figure 5	Influenza/Adeno 1:10	2	Figure_5_Influenza_Adeno_1_10_2.fastq.gz
Figure 5	Influenza/Adeno 1:0.1	2	Figure_5_Influenza_Adeno_1_0.1_2.fastq.gz
Figure 5	Polio/Adeno 1:1	1	Figure_5_Polio_Adeno_1_1_1.fastq.gz
Figure 5	Polio/Adeno 10:1	1	Figure_5_Polio_Adeno_10_1_1.fastq.gz
Figure 5	Polio/Adeno 0.1:1	1	Figure_5_Polio_Adeno_0.1_1_1.fastq.gz
Figure 5	Polio/Adeno 1:10	1	Figure_5_Polio_Adeno_1_10_1.fastq.gz
Figure 5	Polio/Adeno 1:0.1	1	Figure_5_Polio_Adeno_1_0.1_1.fastq.gz
Figure 5	Polio/Adeno 1:1	2	Figure_5_Polio_Adeno_1_1_2.fastq.gz
Figure 5	Polio/Adeno 10:1	2	Figure_5_Polio_Adeno_10_1_2.fastq.gz
Figure 5	Polio/Adeno 0.1:1	2	Figure_5_Polio_Adeno_0.1_1_2.fastq.gz
Figure 5	Polio/Adeno 1:10	2	Figure_5_Polio_Adeno_1_10_2.fastq.gz
Figure 5	Polio/Adeno 1:0.1	2	Figure_5_Polio_Adeno_1_0.1_2.fastq.gz
Figure 6	Plasma	1	Figure_6_Plasma_1.fastq.gz
Figure 6	Plasma	2	Figure_6_Plasma_2.fastq.gz
Figure 6	Urine	1	Figure_6_Urine_1.fastq.gz
Figure 6	Urine	2	Figure_6_Urine_2.fastq.gz
Figure 6	Throat swab	1	Figure_6_Throat_swab_1.fastq.gz
Figure 6	Throat swab	2	Figure_6_Throat_swab_2.fastq.gz
Figure 6	Stool 1	1	Figure_6_Stool_1_1.fastq.gz
Figure 6	Stool 1	2	Figure_6_Stool_1_2.fastq.gz
Figure 6	Stool 2	1	Figure_6_Stool_2_1.fastq.gz
Figure 6	Stool 2	2	Figure_6_Stool_2_2.fastq.gz
Figure S2	Input 1	1	Figure_S2_Input_1_1.fastq.gz
Figure S2	Input 1	2	Figure_S2_Input_1_2.fastq.gz
Figure S2	Input 2	1	Figure_S2_Input_2_1.fastq.gz
Figure S2	Input 2	2	Figure_S2_Input_2_2.fastq.gz
Table 1	Multiplex reagent	1	Table_1_Multiplex_reagent_1.fastq.gz
Table 1	Multiplex reagent	2	Table_1_Multiplex_reagent_2.fastq.gz

Supplemental Figures

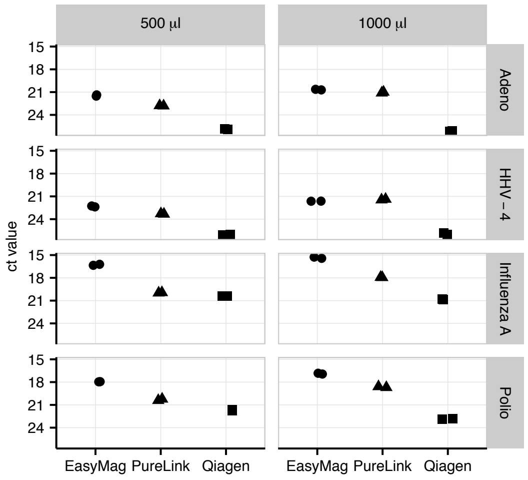

Figure S1: Extraction with the NucliSENS EasyMAG resulted in the highest virus concentrations. Concentrations of four viruses spiked into healthy donor plasma were measured by quantitative PCR after different extraction methods (NucliSENS EasyMAG, PureLink Viral RNA/DNA Mini Kit and QIAamp Viral RNA Mini Kit). Two sample input volumes, 500 or 1000 µl, were tested. Elution volume was 25 µl for all methods. The y-axis is reversed (low ct values equal high concentrations).

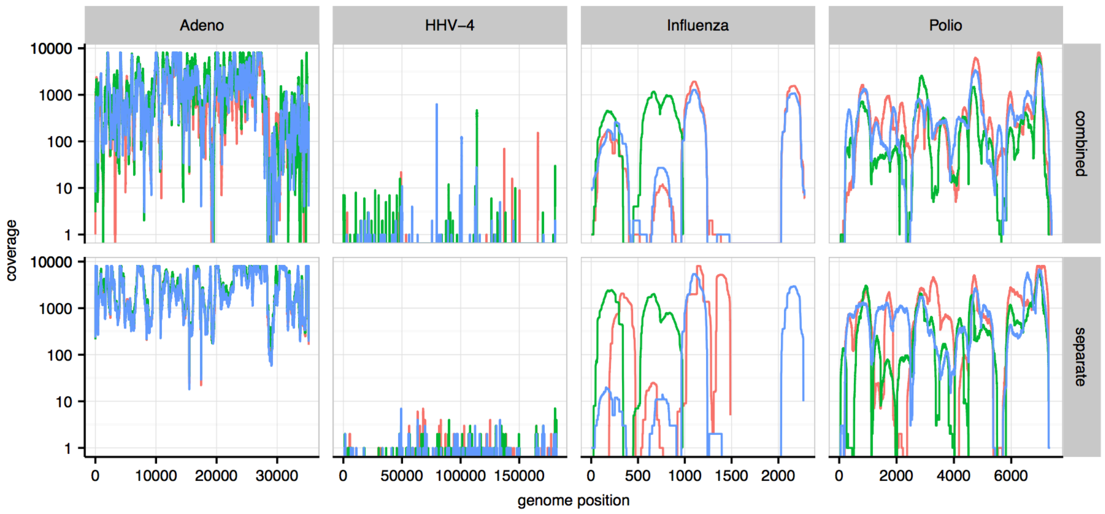

Figure S2: Reads assigned to spiked virus were uniformly distributed along the reference genomes. Plasma samples were spiked with four different viruses (Adenovirus, HHV-4, Influenzavirus, Poliovirus) and processed and sequenced with the "combined" and the "separate" workflow. Coverage plots for all three replicates are shown in different colors.

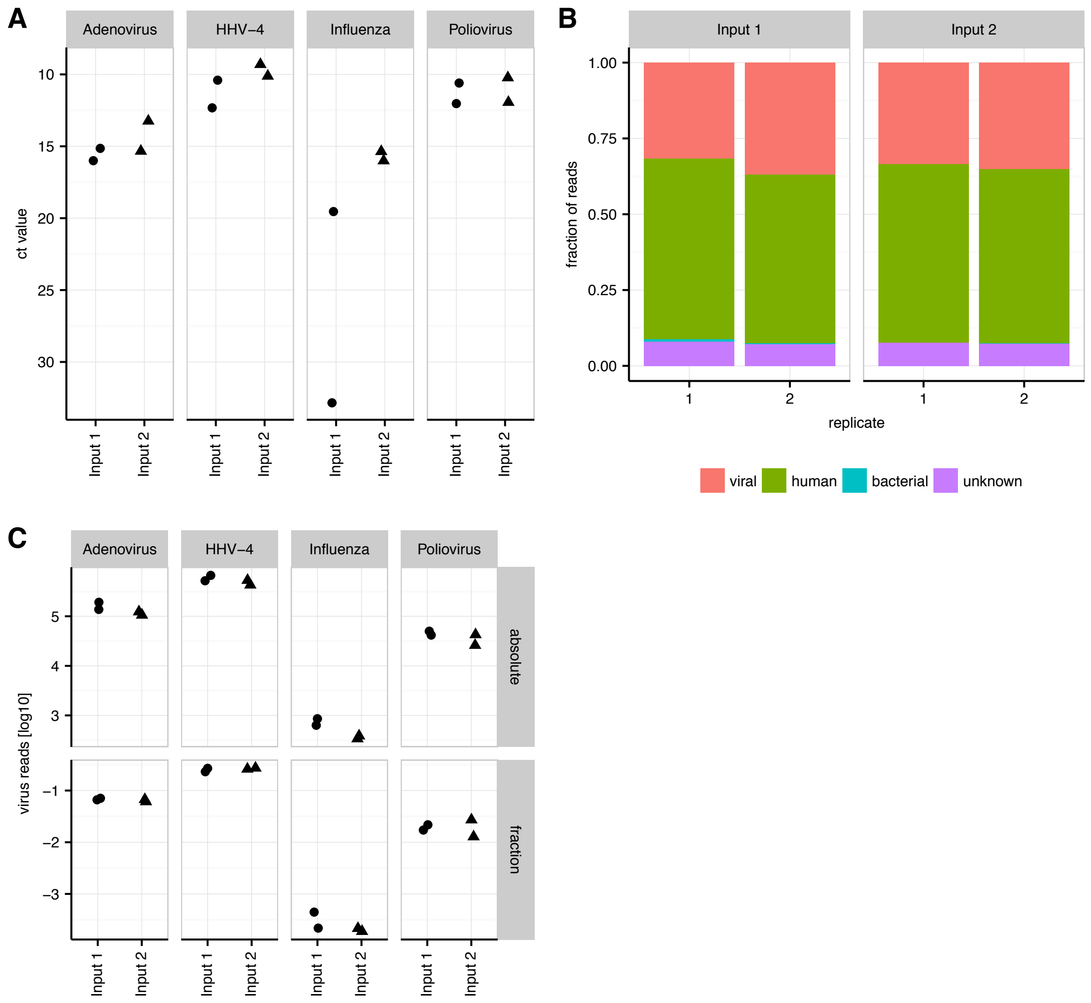

Figure S3: Increasing the PCR input volume had no effect on viral enrichment.

Plasma samples spiked with viruses were amplified with two anchored random PCR protocols, where "Input 1" was 5 µl template in the reverse transcription and 3 µl passed on to the anchor PCR; "Input 2" was 10 µl template in the reverse transcription and 6 µl for the anchor PCR.

A) Virus concentrations measured by quantitative PCR after amplification by unbiased anchor PCR.

B) Distribution of sequencing reads into the different taxonomic categories.

C) Absolute number and fraction of all quality passing reads obtained for each individual virus.

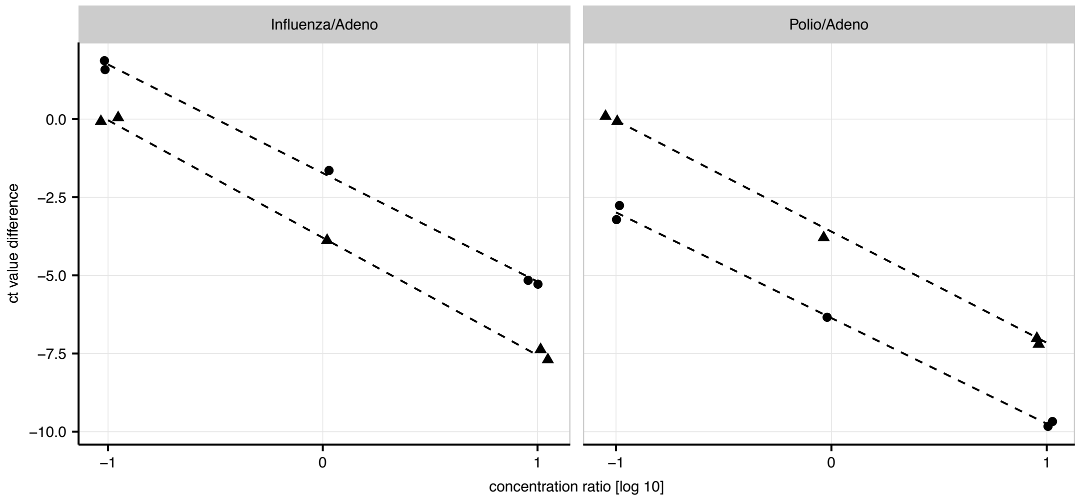

Figure S4: After extraction, viral amplicons were quantified in the eluate and the ratio of spiked viruses was perfectly maintained

Influenzavirus/Adenovirus and Poliovirus/Adenovirus were spiked in healthy donor plasma in different concentrations: at the same concentration for both viruses (log10 concentration ratio = 0), ten times more of one virus (keeping the other virus constant, ratio = 1) and ten times less of one virus (keeping the other virus constant, ratio = -1). After extraction, viral amplicons were quantified in the eluate and ct difference correlated perfectly with the ratio of spiked viruses. Two independent experiments are shown (circles and triangles, respectively). R^2 = 0.99, 0.99, 0.99, 0.99 and p-values = $1.7*10^{-5}$, $1.8*10^{-5}$, $5.3*10^{-5}$, $2.6*10^{-5}$ for Influenza/Adenovirus experiments 1 and 2 and Polio/Adenovirus experiments 1 and 2, respectively.
